# Time to re-think picky eating?: a relational approach to understanding picky eating

**DOI:** 10.1186/s12966-017-0520-0

**Published:** 2017-05-05

**Authors:** Kathryn Walton, Leon Kuczynski, Emma Haycraft, Andrea Breen, Jess Haines

**Affiliations:** 1 Department of Family Relations and Applied Nutrition, University of Guelph, 50 Stone Rd. E, Guelph, ON N1G 2W1 Canada; 2 School of Sport, Exercise and Health Sciences, Loughborough University, Loughborough, UK

**Keywords:** Picky eating, Fussy eating, Bi-directional research methods, Parent-child relationship, Parental feeding practices

## Abstract

**Background:**

Estimates of picky eating are quite high among young children, with 14-50% of parents identifying their preschoolers as picky eaters. Dietary intake and preferences during the preschool years are characterized by slowing growth rates and children developing a sense of autonomy over their feeding and food selection. We argue that the current conceptualization of picky eating defines acts of resistance or expressions of preference (acts of autonomy) by a child as deviant behaviour. This conceptualization has guided research that uses a unidirectional, parent to child approach to understanding parent-child feeding interactions.

**Objectives:**

By reviewing the current feeding literature and drawing parallels from the rich body of child socialization literature, we argue that there is a need to both re-examine the concept and parent/clinician perspectives on picky eating. Thus, the objective of this paper is two-fold: 1) We argue for a reconceptualization of picky eating whereby child agency is considered in terms of eating preferences rather than categorized as compliant or non-compliant behaviour, and 2) We advocate the use of bi-directional relational models of causality and appropriate methodology to understanding the parent-child feeding relationship.

**Discussion:**

Researchers are often interested in understanding how members in the parent-child dyad affect one another. Although many tend to focus on the parent to child direction of these associations, findings from child socialization research suggest that influence is bidirectional and non-linear such that parents influence the actions and cognitions of children and children influence the actions and cognitions of parents. Bi-directional models of causality are needed to correctly understand parent-child feeding interactions.

**Conclusions:**

A reconceptualization of picky eating may elucidate the influence that parental feeding practices and child eating habits have on each other. This may allow health professionals to more effectively support parents in developing healthy eating habits among children, reducing both stress around mealtimes and concerns of picky eating.

## Background

Picky/fussy eating has been defined as an ‘unwillingness to eat familiar foods or try new foods, severe enough to interfere with daily routines to an extent that is problematic to the parent, child, or parent–child relationship’ [1, 2]. Although definitions and measures vary, estimates of picky eating are quite high in the preschool age group, with 14-50% of parents identifying their preschool age children as picky eaters [3, 4].

The early years are characterized as a time for rapid growth and development, with the rate of weight gain peaking by age two and slowing between the ages of two and 5 years [5]. Coinciding with this decreased rate of growth, most preschoolers also experience a decrease in appetite [6]. During this time, children’s appetites can also be quite erratic, with neophobia (initial rejection or avoidance of new foods) and food jags (short term periods of restricted intake) being extremely common [7]. In fact, from an evolutionary perspective, it is actually expected that young children show initial rejection of new foods to ensure that they are not poisonous [8]. Research suggests that with time and repeated neutral exposures, most children will accept new foods [8].

Although changes in dietary intake during the preschool years are influenced by physical changes such as slowing growth rates, children also change psychologically, developing a sense of autonomy, preferring self-feeding, and exercising their own power in food selection. As agents of their own preferences and actions, children may resist eating foods that are unappealing to them. Many of these food selection behaviours that are considered normal in the development of children’s eating habits (i.e., neophobia and food jags) are often considered by parents to be ‘picky.’ In this paper, we argue that the current conceptualization of picky eating defines agentic acts of resistance or expressions of preference by a child as deviant behavior. Similar pathologizing of child agency can be found in the child socialization research, whereby the majority of traditional parent-education and training methods view expressions of autonomy as deviant “noncompliance” and focus on external contingencies such as rewards and punishments as strategies for managing undesirable child behaviour [9].

Parental efforts to influence children’s food choices, such as asking the child to “eat one more bite”, as well as parents’ unrealistic expectations about preschoolers’ dietary intake may lead to increased parental concern and intrusiveness, as well as increased child resistance [10]. This cycle of parental intrusiveness and children’s agentic resistance may cause stressed parent-child feeding interactions characterized by clashes between the parent’s will and the child’s will.

Research has shown that parental feeding practices influence child dietary intake [11–13]. Parental feeding practices encompass a combination of provision and socialization (teaching manners and appropriate eating habits), and are used to manage children’s food intake (i.e. what, when and how much the child should eat) [14]. Traditionally, the specific actions that constitute parental feeding practices have been used to categorize a parent’s overall feeding style. Specifically, parental feeding practices have been categorized on the type of control the parent exerts over the feeding interaction [15]. Directive control, or coercive control, refers to feeding practices that are highly controlling such as pressuring the child to eat, restricting certain foods or food groups, and the use of food as a reward [16]. Non-directive control refers to feeding practices that aim to influence child eating via less direct methods, such as monitoring the child’s intake, modelling of desired eating habits and food intake and gentle encouragement [15]. Directive feeding practices have shown to be associated with poorer dietary intake; by focusing children’s attention on external cues, such as parental rewards or pressure, these directive feeding practices could undermine children’s ability to self-regulate their dietary intake [15].

The use of directive feeding practices may develop from parental concern about their child’s dietary intake or lack of knowledge about age-appropriate eating behaviours. This concern may be exacerbated because of parents’ perception that their child’s refusal of certain foods constitutes disobedience or noncompliance such that parents’ focus becomes compliance to authority rather than the promotion of healthy or diverse food choices. We argue that by helping parents and practitioners to recognize and respect children’s agentic responses as an aspect of the normal etiology and development of young children’s eating habits, parental concern surrounding child eating habits may subside, allowing for more relaxed and enjoyable feeding interactions.

Conceptualization of picky eating as a deviant or non-compliant behaviour has guided research, which is based on a parent-centered unidirectional perspective (i.e. exclusive focus on what the parent believes, wants or does) to understanding parent-child feeding interactions [17]. However, parenting - including parental feeding practices - is, in part, a response to child characteristics and behaviours, as much as child eating habits are, in part, a response to those of the parent [18]. Further, when thinking about the parent-child relationship, researchers are often interested in understanding how members in the dyad affect one another: do the parent’s feeding practices influence the child, or does the child’s behaviour influence the feeding practice used by the parent [19]? While many researchers tend to interpret associations between parent and child measures as parental influence on the child, the reality is that parenting influences child eating habits and child eating habits influence parenting in non-linear ways. Thus, we need to use bi-directional approaches to correctly understand parent-child feeding interactions. Moving our thinking about picky eating from a, unidirectional (parent → child) perspective, to that of a bi-directional (parent ← → child) perspective where parent and child co-actions create expectations for feeding interactions, may be the first step in elucidating the relationship.

The dialectical causal concept of co-action implies that the feeding outcomes are the joint product of the agentic actions of both parent and child. Co-actions can be cooperative (i.e. parent and child negotiate an outcome is mutually acceptable and allows the goals of both to be upheld during eating), or uncooperative (i.e. parent and child resist each other during mealtimes, and do not produce mutually acceptable outcomes) [19]. This shift to a bidirectional perspective will allow researchers to understand the influence that parental feeding practices have on child outcomes and vice versa. Such an understanding will allow health professionals to more effectively support parents in developing healthy eating habits among children, thereby reducing stress around mealtimes and concerns of picky eating.

It should be noted that there have been some important, pioneering, steps toward a bidirectional perspective on parent-child feeding interactions. In their review of food parenting practices, Vaughn and colleagues [16] discuss the idea of ‘autonomy support’ and parent feeding practices that can nurture a child’s ability to self-regulate, including providing children with choice, discussing rules and boundaries surrounding food and emotional support during feeding interactions. Using a discordant twin analysis, Harris and colleagues found that mothers vary their feeding practices for twin children who differ in their ‘food fussiness’; mothers reported using more pressure to eat and more rewards with the fussier twin in comparison to the less fussy twin [20]. Vandeweghe and colleagues [21] also found a differential effect on strategies used to get children to try disliked vegetables, whereby children high in reward sensitivity responded strongest to rewards, and children low in reward sensitivity respond best to verbal encouragement. However, while the few prospective studies examining parental feeding practices and child eating habits are useful for assigning direction to associations with more confidence, they often explore the interaction from a unidirectional (parent → child) [22, 23] or (child → parent) [24] perspective.

Given the need to both re-examine the concept and parent/clinician perspectives of picky eating, the objective of this paper is two-fold: 1) We argue for a reconceptualization of picky eating whereby children’s resistance during eating is considered as children’s agency in expressing eating preferences rather than categorized as compliant or non-compliant behaviour, and 2) We advocate the use of bi-directional relational models of causality and appropriate methodology to understanding the parent-child feeding relationship.

## Discussion

### Re-conceptualizing picky/fussy eating

Although research has suggested that picky eaters often have reduced energy intake [2, 4, 25], eat fewer fruits and vegetables [2, 26, 27] and have lower intakes of dietary fibre [27], the impact on child growth has been mixed, with some studies suggesting no impact [6, 28], while others suggest a higher risk of being underweight [3, 29] or overweight [30]. The unidirectional approaches used in the current body of literature, including the definition of picky eating itself, limit the interpretation of this research. Are the children’s dietary intakes suboptimal due to organic disease [28] or in extreme cases, a primary psychiatric disorder (i.e. avoidant restrictive food intake disorder) [31], or because the feeding practices used by their well-intentioned parents purse compliance to their unrealistic wishes as a goal? We argue that labelling a child as a “picky”, “fussy” or “noncompliant” itself may contribute to difficult feeding interactions because such labels pathologize what may be normal variations in children’s feeding preferences and increase parent and child stress. This increased stress may lead to either an escalating cycle of discordance between parent’s and child’s will or a situation in which parents withdraw from their efforts and cater to the child’s will, allowing poor dietary habits to prevail. Briefly, it should be noted that while food neophobia and short-term food jags are very common, children who eat an extremely limited range of foods for long periods of time, who do not accept foods back after food jags (which results in intake being further restricted), and those who refuse entire categories of food textures or nutrition groups may require more support from a dietitian and an in-depth assessment of the parent-child feeding interaction. In cases where suboptimal intake has impacted the child’s growth (i.e. dropping percentiles), as identified using age- and- sex-appropriate growth charts, more intensive therapy, in addition to the discussion of and support focused on parent and child roles during eating, may also be warranted. The majority of parent-defined “picky” eaters do not fall into these definitions; however, all parent concerns related to their child’s dietary intake should be explored to reduce anxiety and the potentially negative feeding practices that result from this concern.

In the child socialization literature, researchers have proposed that resistance can be a positive aspect in the social development of a child by providing a context for children to assert their autonomy within the parent-child relationship and develop the social skills with which to appropriately express their autonomy in a socially acceptable manner [32, 33]. Within the realm of feeding, we can understand this as the process by which children learn socially appropriate ways of social interaction, including table manners, and explore the food in their environment. Rather than labelling children as “noncompliant” or “picky eaters”, parents can be encouraged to re-conceptualize children’s actions during mealtimes as agentic preferences, some of which are discriminating regarding non-nutritional qualities such as taste, texture, presentation, and familiarity. Rather than focusing on control strategies for obtaining compliance to parental preferences, we can focus parent attention on fostering children’s healthful intakes and expanding their children’s culinary horizons through non-directive feeding practices such as modelling, participatory education, and setting positive relational contexts for meals. In addition, it may be constructive for parents to adopt accommodation rather than exact, immediate compliance as an acceptable outcome of feeding interactions [9, 19]. Accommodation implies that the child acknowledges that the parent has been heard and that the nature of the cooperative response is a creative, negotiated outcome that reflects the joint agency of parents and children [9, 19]. Accommodating the child’s agency and accepting the child’s accommodations of the parent’s efforts may ward off the negative directive feeding practices by allowing children’s eating behaviours and food preferences to evolve naturally in coactions with their parent. Among preschoolers, accommodation may take the form of partial acceptance of the parent’s request such as trying or tasting the food served or by negotiating the amount, or nature (e.g. not including the sauce) of the food. Parents can also align conversations with their preschoolers that allow for collaboration, for example, rather than asking children to “eat three more bites”, parents could ask their children’s perspective regarding their internal state of satiety, such as “does your tummy feel like it has more room”, or the child’s experience of the food, “Does this taste okay to you?” Research supports that this respect for autonomy leads to positive child outcomes; in their study of Mexican-American mother-child dyads, Hays and colleagues found that mothers who used explanations and minimal pressure during feeding had children who displayed greater understanding of the role of food in maintaining health [34]. Further, discouraging child agency encourages children to eat past their natural satiety points, prohibiting their ability to self-regulate intake [35, 36]. Finally, the children of parents who model healthful eating habits have been reported to be less ‘fussy’ [37], more likely to try disliked vegetables [21] and have higher intakes of fruits and vegetables [38].

Implied in our re-framing of the view of picky eating, is the distinction between the parent’s desire for child compliance to their own immediate, short term goals (getting the child to eat the meal in front of them) and the long-term goal of fostering children’s internalization of more diverse and healthy food preferences. Parents use power-assertive methods, i.e., controlling or coercive feeding practices, to achieve short term objectives (such as using food as a reward to get a child to eat his or her vegetables or pressuring the child to eat “just one more bite”), but use explanations and modelling when longer term objectives are the goal (such as explaining how milk helps grow strong bones and teeth, or modelling polite table manners during meals) [34, 39]. Labelling a child as a ‘picky/fussy’ eater may cause parents to focus too heavily on short-term objectives of getting the child to eat. By encouraging parents to re-frame picky eating as an appropriate act of agency, we may be able to foster mutually reciprocal parent-child relationships that are more conducive to positive feeding interactions both in the short and long term. In fact, research has suggested that pressuring children to eat new foods may cause dislike for the food [40].

### Understanding parent-child dynamics in feeding situations: Moving from unidirectional to interactional to dialectical conceptions of the feeding process

Our reconceptualization of feeding emerges from changes that have occurred in the understanding of socialization processes where there has been an increasing recognition of the role of children’s agency and influence in parent-child dynamics [19]. Historically, the unidirectional framework, on which much feeding research is based, was challenged many years ago by Bell’s 1968 demonstration that correlations between parenting behaviors and children’s outcomes can be plausibly interpreted as the effects of children’s temperament on parental practices [41]. Bell and Harper’s 1977 control process model was an early bidirectional approach to explain child effects using an interactional model where changes in parent and child behaviors emerge from reciprocal exchanges and reactivity in parent and child behaviors over time [42]. Bell and Harper [42] suggested that parents and children establish a range of appropriateness for their interactions, including the frequency and intensity of behaviours that can be tolerated by the other. When one partner in the dyad exceeds this range at either the upper or lower level, the other takes action to bring the others behavior into their own range of tolerance [43]. For example, parents may have an expectation about what and how much food the child should eat, as well as the table manners the child is expected to display. If a child acts at the lower level of this expectation, and does not eat his vegetables, the parent may react to increase their intensity of a specific feeding practice to increase the intake of the vegetables (i.e. pressure the child to eat). Thus, parental pressure is an effect of the child’s noncompliance. In an extension of the control process model from the child’s perspective, if the pressure used by the parent exceeds his or her tolerable range, he or she will act so as to evade or stop the parent’s pressure [42]; the child’s resistant actions are an effect of the parent’s behaviors. Parent and child will escalate their attempts to bring the other’s behavior under control until one succeeds, thus completing the feeding interaction and forming a basis for the next. This example highlights the typical parent-child interaction that occurs with children that are labelled as “picky eaters.” Although the control process model is useful for understanding detrimental processes that may occur in feeding interactions, it is limited in that it focuses on the reciprocal behavioral and emotional reactivity of parents and children as well as linear behavioral control tactics rather than constructive processes involving parent and child agency. More recently this interactional or behavioral approach to bidirectional influence has been challenged by dialectical or transactional models of bidirectional processes.

### A dialectical approach to the bi-directional parent-child relationship

Interactional models of parent-child processes examine parents and children reciprocally exchanging behaviors or reacting to each other emotionally and there is little consideration of parent or child agency. In contrast, in new dialectical models, both parent and child are understood to have agency. The main facets of the dialectical approach are that the parent-child relationship is based on social transactions rather than interactions, meaning that parents and children engage in mutual meaning-making, rather than just reacting to one another’s behaviours [19]. Applications of the dialectical model to the phenomenon of parent-child feeding and eating can be understood in three distinct but interrelated processes: transactional, relational, and bilateral processes of agency, influence and power [19].

The transactional model was first developed by Sameroff [44] and presented the idea that the child alters his parent and is, in turn, altered by his changed parent. The model focuses on qualitative transformations that occur as parents and children respond to and make sense of the contradiction represented by the other person’s actions. For example, parents have their goals for children such as teaching children about healthy eating or wanting them to eat the foods served, and in turn children have unique likes and dislikes as well as their own ideas about their eating and what the parent’s behavior means to them.

Kuczynski and De Mol (2015) built upon this model with their social relational theory [19]. A distinctive feature of the social relational model is that it places equal emphasis on the perspectives of both the parent and the child. Both parents and children are agents who maintain their own autonomy in the meaning of the feeding relationship by interpreting messages from the other, and acting on those meanings; a child’s refusal to eat certain foods may be seen as their attempt to show autonomy over their eating, but also may communicate to parents that they did not appreciate the pressure to eat [19]. During the preschool years, children develop increasingly assertive and skillful strategies for overtly challenging parents [33]. This could be why battles surrounding picky eating are often strongest during this age group. As children age, they use more covert strategies to achieve their goals while evading confrontations in effort to both preserve their own agenda and protect their relationships with parents despite pursuing their own goals [45].

The social relational model also emphasizes the importance of the long-term parent-child relationship as a context for understanding how parent and child agency is expressed as well as the dynamics of bidirectional influence occurring between parents and children. Despite differences in their perspectives and goals, the parent and child are bound by a mutual relationship in which they both have a stake and thus, try to accommodate each other’s perspectives [19]. Each feeding transaction incorporates past and anticipated future feeding interactions, making up the relationship context for feeding [19].

In addition, social relational theory proposes that the parent child relationship is complex. On a daily basis bidirectional transactions between the parent and child engage different domains of the relationship including attachment, authority and intimacy [19]. Power dynamics are important to consider in each of these domains as parents and children co-act to reach a mutual understanding. In the authority domain, parents use their asymmetrical power to influence children’s eating behaviors and bidirectional dynamics can take the form of conflict and coercion or mutual accommodation of the others’ agency. In the attachment domain, power is complementary whereby the child seeks to be fed and the parent provides; bidirectional dynamics can take the form of mutual responsiveness or non-responsiveness to each other’s needs. In the intimacy domain, power is relatively equal and allows parents and children to share and co-create meaning during opportunities such as mealtimes set up for mutual enjoyment. Interactions where one imposes or rejects meaning is destructive to intimacy.

The conflicts that arise in each of these domains may go beyond the immediate situation and may influence how parents and children understand the nature of their relationship. For example, within the authority domain, when a parent refuses to ‘short order cook’ for the child after the child refuses to eat the meal served, the child will learn that the parent’s power prevails when conflict arises. Within the attachment domain, parents’ responsiveness and non-responsiveness to children’s requests for food will have implications for the child’s sense of security in the relationship, as well as on the child’s overall eating habits. In fact, research has shown that in comparison to high parental responsiveness, the low responsiveness shown in neglectful parenting is associated with significantly higher odds of obesity among young children (OR = 1.56, 95% CI 1.14-2.14) [46]. It is argued that in this context, children do not gain the self-regulation skills that form the basis of healthy eating [47]. Finally, within the intimacy domain, mealtimes can serve as a time to construct mutual closeness, cooperation and conversation in the context of feeding, and thus promote an anticipation of that feeding is a context for enjoyable interaction.

The implication of the dialectical consideration of context is that feeding is not just about nutrition, it is about relationships. What happens in the feeding situation has implications for other domains of the relationship including authority, attachment and intimacy and each context has implications for the dynamics of feeding interactions. When measuring feeding interactions from bi-directional perspective, we need to consider the relational climate (broader family context) within which the feeding interaction occurs.

In summary, the dialectical approach focuses on mutual meaning making between parent and child. Focusing on three domains of interaction, conflicts within these domains give rise to new meaning making and the basis for the next parent-child interaction. From this approach, it is important to view both the parent and child perspectives simultaneously rather than as separate entities.

### Applications of the dialectical model to current practice

While not empirically tested, Ellyn Satter’s ‘dynamics of feeding’ principle guides some of the professional advice given to parents about feeding their children. This model views the parent as responsible for ‘what’, ‘where’ and ‘when’ the child eats, and the child as responsible for ‘whether’ and ‘how much’ he eats [48]. This model fits well within the dialectical approach as it recognizes that parents and children are equally co-acting agents and allows parents and children to come to a place of mutual understanding and trust in their feeding transactions. Parents are responsive to the child’s needs and, in turn, the child can show autonomy over his or her eating habits. Conflict that does arise within this model allows both parent and child to adjust their expectations for future transactions, allowing mealtimes to become a time to build mutual intimacy and enjoyment.

From a research perspective, we are still in the pioneering stages of considering bi-directionality in the parent-child feeding relationship. Kuczynski and Parkin [49] provide a helpful guide for adapting feeding research to a dialectical model of bi-directionality. First, parents and children need to be viewed as equal agents. By the very nature of this, researchers are suggested to challenge linear thinking. While linear outcomes are often a “happy ending” for research projects, continuous change and new synthesis are expected outcomes of socialization research where parents and children continually learn more about and adapt their relationship through transactions [49]. Re-conceptualizing picky eating to consider child agency rather than dichotomizing eating behaviour as compliant and non-compliant, is an important first step in moving towards this dialectical perspective. Second, rather than thinking about parents and children as individuals engaged in discrete social interactions, Kuczynski and Parkin suggest thinking of them as engaging in transactions in a long-term relationship context [19, 49]. Third, rather than focusing on direct causation between behaviours (i.e. parent feeding practices) and outcomes (i.e. child dietary intake), it is important to search out conditions associated with change [19]; contradictions present opportunities for change and potential intervention [49]. These steps were designed by Kuczynski and Parkin to provide personal resolutions for researchers transforming into transactional research [49]. Moving forward, we suggest two practical implications of these resolutions in future research: 1) researchers need to observe feeding interactions between parents and children and 2) where feasible, feeding interactions need to be observed longitudinally, or at a minimum, consider parents’ and, when age appropriate, children’s long term goals associated with the relationship. In the next section, we provide suggestions for methods that are well suited towards the idea of child agency and a dialectical model of bi-directionality.

### Directions for future research

Future research should consider the bi-directional relationship that encompasses parent-child feeding interactions from a dialectical approach. Although understanding how parent and child behaviours influence each other is important, we argue that it is also important to think about how parents and children make meaning from these interactions to either change or sustain behaviour. Mealtimes are a continuation of other parent-child interactions that occur throughout the day and so feeding and accepting what is being fed might not be about the food item or compliance to the parent, but could be influenced by meaning making around other aspects of family life. Using a dialectical lens will allow future research to underscore the complexity of what is happening during family mealtimes. Introducing a dialectical approach to understanding parent-child feeding practices is an important step in acknowledging this complexity.

Mixed-method longitudinal studies are the best method with which to explore this. Observational methods provide an objective measure of both parent and child behaviours and a longitudinal design will provide insight into how the feeding relationship develops and changes over time as the parent-child relationship itself develops and changes. Further, observational methods account for the context within which the feeding interaction occurs. While one study [50] found associations between parent reported feeding style, observed feeding practices and the emotional feeding climate, no other associations have been found between observed and reported parental feeding practices [51]; observations capture practices that may be either too complex, decontextualized or habitual for parents to report using rating scales as the sole method. Such methods also allow researchers to understand child agency within the context of the feeding interaction. Further, when thinking about intervention research, observational methods designed to consider parent-child feeding transactions provide a novel opportunity to tailor feeding interventions to the specific parent-child dyad while considering the broader relationship within which feeding occurs. For example, video-taped meals could be used as a novel counselling technique whereby the practitioner can provide specific input and feedback on the interactions observed.

Qualitative interviews also provide a unique opportunity to support the objective measures collected through observation by allowing parents to reflect upon or provide more context towards the complexity of parenting, child feeding, and family life. Qualitative methods can be thought of as the cognitive counterpart of observational methods in that both are concerned with interactions and experiences as they occur in natural environments [52]. This method may be helpful in clarifying parent and child cognitions that make up the experience of feeding as well as the meanings, attributions and goals that parents and children construct from those experiences. Interviews and event diaries may be constructed from a bi-directional framework whereby parents are asked to report on the relational ways in which mealtimes are structured [53]. For example, using the critical incidents technique [54], parents can provide narrative descriptions of their actions during meals by commenting on specific instances during meals in which they were influenced by their child and in turn, their response to these influences [53].

## Conclusions

Throughout this paper we have presented an alternative conceptualization of picky/fussy eating whereby the child is granted agency in their eating habits, as well as for a dialectical perspective of the parent-child feeding relationship whereby both parents and children have agency to understand their co-actions in the context of their greater relationship. In combination, we believe that this reconceptualization of picky eating may strengthen our understanding of the parent-child feeding relationship, elucidating the influence that parental feeding practices and child eating habits have on each other. Finally, from an applied standpoint, in addition to promoting appropriate parental expectations and non-directive feeding practices, health professionals are encouraged to challenge the labelling of ‘picky eater’ and instead, focus conversations with parents around their expectations of children’s eating and the interactions they have with their children during mealtimes.
